# In Vitro and In Vivo Functional Viability, and Biocompatibility Evaluation of Bovine Serum Albumin-Ingrained Microemulsion: A Model Based on Sesame Oil as the Payload for Developing an Efficient Drug Delivery Platform

**DOI:** 10.3390/ph16040582

**Published:** 2023-04-12

**Authors:** Atiaf Rhyaf, Hala Naji, Hassan Al-Karagoly, Salim Albukhaty, Ghassan M. Sulaiman, Abdulaziz Arif A. Alshammari, Hamdoon A. Mohammed, Majid Jabir, Riaz A. Khan

**Affiliations:** 1 Department of Pathology, College of Veterinary Medicine, University of Al-Qadisiyah, Al-Diwaniyah 58002, Iraq; 2 Department of Internal and Preventive Medicine, College of Veterinary Medicine, University of Al-Qadisiyah, Al-Diwaniyah 58002, Iraq; 3 Department of Chemistry, College of Science, University of Misan, Maysan 62001, Iraq; 4 College of Medicine, University of Warith Al-Anbiyaa, Karbala 56001, Iraq; 5 Division of Biotechnology, Department of Applied Science, University of Technology, Baghdad 10066, Iraq; 6 Department of Medicinal Chemistry and Pharmacognosy, College of Pharmacy, Qassim University, Buraydah 51452, Saudi Arabia; 7 Department of Pharmacognosy and Medicinal Plants, Faculty of Pharmacy, Al Azhar University, Cairo 11371, Egypt

**Keywords:** biocompatibility, bovine serum albumin, LD_50_, microemulsion, functional viability, nanomedicine, nanoparticles, drug delivery, liver biomarkers, hepatic functions, biological marker, sesame oil, toxicity, cytotoxicity

## Abstract

Combination of bovine serum albumin with microemulsions as constituting ingredient biopolymer has long been regarded an innovative method to address the surface functionalization and stability issues in the targeted payload deliveries, thereupon producing effectively modified microemulsions, which are superior in loading capacity, transitional and shelf-stability, as well as site-directed/site-preferred delivery, has become a favored option. The current study aimed to develop an efficient, suitable and functional microemulsion system encapsulating sesame oil (SO) as a model payload towards developing an efficient delivery platform. UV-VIS, FT-IR, and FE-SEM were used to characterize, and analyze the developed carrier. Physicochemical properties assessments of the microemulsion by dynamic light scattering size distributions, zeta-potential, and electron micrographic analyses were performed. The mechanical properties for rheological behavior were also studied. The HFF-2 cell line and hemolysis assays were conducted to ascertain the cell viability, and in vitro biocompatibility. The in vivo toxicity was determined based on a predicted median lethal dose (LD_50_) model, wherein the liver enzymes’ functions were also tested to assess and confirm the predicted toxicity.

## 1. Introduction

Microemulsions (MEs) are transparent, isotropic liquid systems that are spontaneously formed when water, surfactant, and oil constituents are combined [1]. MEs can be formed instantaneously without the need for high-shear equipment. Furthermore, the MEs have distinctive characteristics, such as, small particle size of 100 nm (or less), low viscosity, single optical property, and thermodynamic stability. The use of MEs have been expanded to several biomedical and pharmaceutical applications, including dosage forms preparations of parenteral, oral, pulmonary, and ocular formulations, as well as for topical drug delivery formats [2,3].

Nanotechnology has made significant advances over the years. Nonetheless, the nanostructures fabrication, preparative processes, and products’ controls, including intended and specified selective payload delivery through them, and other biomedical applications of nanostructures, still today remains, to a certain extent, elusive in terms of transitional and shelf-stability, and the functional efficiency. To overcome these challenges, countless efforts have been made over the past decades. However, several involved factors were needed to be addressed, including surface coating and functionalization through the surface attachments of the molecular/biomolecular tag and structural addendums, particles’ agglomeration, size uniformity, toxicity control/reduction, biocompatibility, biodegradation, and chronological, as well as, tissue-specific targeting [4,5]. Inherently, the nanoparticles have a high surface area-to-volume ratio, which results in rapid absorption of plasma proteins upon intravenous delivery, passage out of the reticuloendothelial system (RES), and protein corona formation over longer duration stays for certain types of nano-entities. To improve nanostructures’ stability, functional feasibility, and poly-dispersity, the natural and synthetic polymers and macromolecular biopolymers attachments and coating incorporations, have been employed. Their inclusions as constituents/ingredients in different forms for various applications role, have been frequently applied. The polymers and biopolymers included poly(vinyl alcohol) (PVA), poly (D, L-lactide-co-glycolide) (PLGA), dextran, poly(ethylene glycol) (PEG), chitosan, bovine serum albumin (BSA), and several other bio-polymeric products, though the most commonly used biopolymers were BSA, and human serum albumin (HSA) [6,7,8,9,10]. A plethora of advantages have been attributed to the use of BSA during the preparation of ME (microemulsion), which included improved biocompatibility, biodegradation, improved safety, transitional and shelf-life stability, introduction of non-toxicity through prevention of the toxic effects of the payload owing to its encapsulation, non-IgE reaction, reduction/removal of immune adverse effects, ability to dissolve in water, easy availability and cost-effectiveness of the biopolymer, as well as ease of systemic circulation clean-up. Moreover, the BSA seemed to also serve as a steric barrier to protein adsorption, resulting in reduced RES uptake by the macrophages, which is ultimately associated with an increase in the serum/biological half-life of the final nano product [11]. According to a new 2022 study, conducted by Zwain, et al. [12], BSA is often used in drug delivery owing to its cost-effectiveness, feasibility of preparing the formulation, and high safety profile. It is also more tolerated as compared to other natural polymers/biopolymers, which is because BSA is a biodegradable, biocompatible, non-toxic, and non-immunogenic biopolymer, and so it has been frequently employed as a colloidal delivery system. In fact, the BSA is a first preferred choice for the drug delivery purpose. Additionally, because the BSA molecules contain drug-binding sites, the generated carrier can be loaded with a range of drugs, and other bioactive compounds. The surface of BSA molecules also have several functional groups (carboxylic and amino groups in abundance), which enables the covalent attachment of cell-targeting substances, e.g., folate, transferrin, monoclonal antibodies, cationic polymers, and several others, which makes the carrier specifically site-directed for the intended site-specific delivery [13]. This method has improved the existing approach for high-incidence, target-specific delivery. Moreover, the BSA has a blood half-life of approximately 19 days, so it can also provide sustained delivery over a longer period of time with high loading of the drug onto the carrier [14].

Surface functionalization is a prominent conjugation approach for targeting the ligands to specific locations and molecular sites of cellular and tissue-specific requirements. The conjugates have the ability to control the intelligent binding of the MEs to particular cell types, or physiological states and conditions based on their biochemical paradigm and availability of the molecular target(s) as well as the cellular ligands [15]. The activation of the surface to combine through one or more cell ligands seemed to induce a certain kind of artificial/circumstantial decision for attachments to enable the payload delivery. This method has been widely employed in a variety of settings [16]. The lack of a suitable surfactants for the production of MEs, particularly oil-in-water (O/W) kinds, is the primary drawback towards achieving this. Incorporating the BSA into the ME is a novel way of overcoming these issues, since it contains several functional groups, including carboxylic (-COOH), amino (-NH_2_), and hydroxyl (-OH) functional entities [17]. Additionally, herbal-based MEs have also recently been developed for use in biomedical applications, due to their high bioavailability, and excellent biocompatibility and biodegradation in the system. Sesame oil, an herbal medicine and a food, is used in the tropical regions of the world due to presence of high levels of unsaturated fatty acids in it, which included oleic and linoleic acids, together with some proteins, minerals, lignans, tocopherol, and phytosterols. Sesame (*Sesamum indicum* L.) seeds are composed of 45% to 50% lipids, 5% to 6% moisture, 10% to 15% carbohydrates, 5% to 6% ash, 4% to 5% fiber, and 15% to 20% proteins [18]. It has been shown that sesame oil has many health benefits through increased levels of plasma tocopherol, and increased vitamin E activity, which reduces the mechanisms of aging and malignancy as well as heart diseases [19]. A recent study published by Wu, et al. [20] found that lycopene bio-accessibility was around 25% when lycopene-loaded nanoemulsions were made by a high-pressure homogenization technique using sesame oil as the oil phase, and lactoferrin as the emulsifier. However, only a few studies addressing the use of extended surfactants from plants’ oils extractions have been reported over the past 10 years. 

The current study attempted to fabricate an ME carrier system based on natural herbal, sesame oil, as the oil phase, incorporating BSA as the constituent ingredient of the ME. For the use of designated ME in medicine and pharmaceuticals, such as drugs and gene deliveries, and development of probable biosensors, as well as nano-ligands for cancers treatment, it is extremely important to investigate the toxicity, and biocompatibility of the produced system, while it is known that certain drugs, or other payloads may, or may not, be toxic, toxic enough to cause harm. For instance, sesame oil, which is by far the least toxic material among the edible oils and similar category of oily produce, is frequently used as part of the foods in larger quantities by a substantial quarters of the world population. In this context, for the testing the final toxicity of the prepared system, its payload, and encapsulating materials, it seemed pertinent to test the carrier’s formulating ingredients, and the toxicity, and biocompatibility of the carrier itself. Most toxicity studies have been conducted in the in vitro conditions, because the process, firstly, is feasible to set-up, and secondly, easy to conduct, control, and interpret the findings, as compared to the in vivo trials. The in vivo trials, where many factors of a significant and insignificant nature are seemingly at play, and whose role in the toxicity test is uncertain. It is simple and efficient, for the most part, to proportionally investigate the toxic effects of such materials by carrying out in vitro toxicity studies.

Therefore, during the current study, the in vitro and in vivo toxicity and biocompatibility of the suggested ME-based carrier system were investigated. A hemolysis assay, and cell viability tests were used as in vitro standard tests to determine the biocompatibility, and toxicity of the suggested ME. To evaluate toxicokinetics, the median lethal dose (LD_50_), and the mean effective single dose (ED_50_) were used as part of the standard tests. The effective dose, or ED_50_ value, describes the concentration at which 50% of the defined effect takes place, when a given dose is used in a laboratory set-up. The LD_50_ value described the dose that killed 50% of the laboratory’s model test animals. Mice were used in animal-based experiments to determine the LD_50_ and ED_50_ values of the formulation. Thus, the procedure also assessed the LD_50_ assay for the developed ME as part of an in vivo toxicity test.

**Figure 1:**
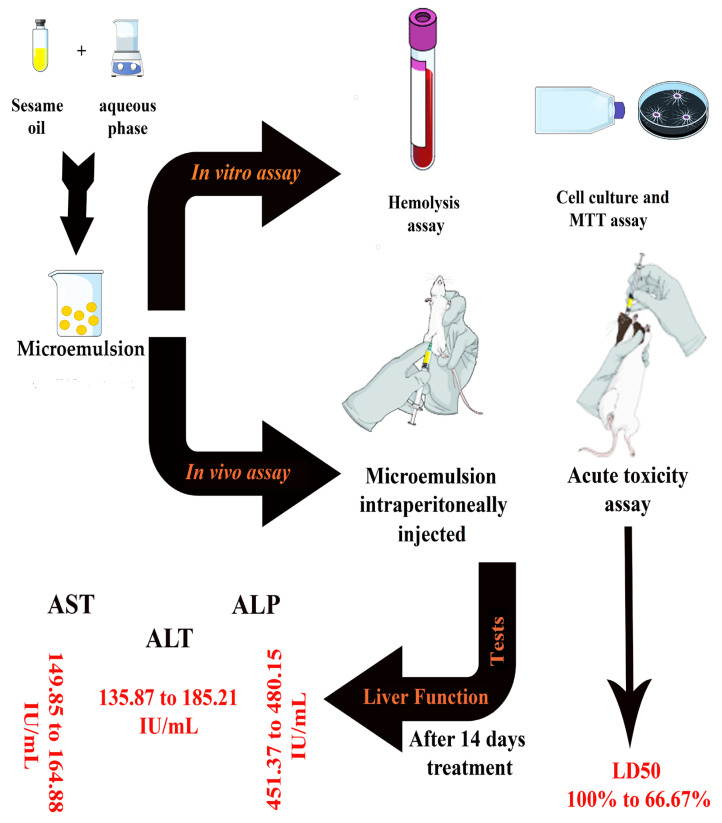
A depiction of the experimental research design and steps during the study.

The liver is an organ with a high capacity for regeneration. As part of the in vivo toxicity study, the alanine aminotransferase (ALT), aspartate aminotransferase (AST), and alkaline phosphatase (ALP) levels were measured during the current study’s set-up. Additionally, the prepared and selected oil/water (O/W) ME was characterized using FT-IR spectral analysis, the DLS technique, UV-Vis spectroscopy, FE-SEM, and rheological behavior analysis was carried out. A plan of work, experimental design, approach and steps flow chart are provided in Figure 1.

## 2. Results and Discussion

### 2.1. Formulation of Optimal Microemulsion Carrier

The aqueous (water, surfactant, and co-surfactant), and oil phases (composition of sesame oil) were the two main phases required for the production of MEs. Because of their low surface tension, glycerol was chosen as a co-surfactant. Since glycerol is highly soluble in water, its incorporation into the surfactant layer can reduce the polarity of the water, thereby increasing the interfacial fluidity, as also previously demonstrated [21]. It has been shown that glycerol can promote the merging, and incorporation of more water because it has the potential to increase the surfactant’s elasticity [22]. Tween-80 was chosen as a surfactant in the study, owing to its high hydrophilic–lipophilic balance (HLB value = 15), biocompatibility, and low toxicity. A liquid agent capable of emulsifying fat and oils was also developed by combining Span-60 (HLB = 4.3) and Tween-80. The findings demonstrated that the stability of the formulation was achieved through dissolving low-HLB-value Span-60 in the oil phase, and high-HLB-value Tween-80 in the water phase. As a result, the kind of surfactant and co-surfactant, as well as their concentrations, which were important criteria in the ME preparation, were followed. The HLB value, which is the ratio of hydrophilic to lipophilic groups, effectively indicates whether a surfactant will promote an oil-in-water emulsion, or a water-in-oil emulsion. This can be estimated by calculating the percentages of the molecular weights of the hydrophilic and lipophilic structural portions of the surfactant molecule. As a result, several sesame oil, water, surfactant, and co-surfactant ratios were tested to identify the optimal O/W ME formulation (Table 1). Span-60 was omitted from the first six formulations of MEs. The experimental results on these formulations demonstrated separations of the aqueous and organic phases. The results revealed that Span-60 played a significant role in the formulation’s stability, which was validated by other studies also [23,24]. 

Although Tween-80 was used as a surfactant in four different formulations constituting the Span-60 (7–10 formulations) also, the prepared MEs seemed to be slightly cloudy, and their phases separated after 7 to 14 days. This could have occurred due to the lack of the surfactant mixture (Tween-80 and Span-60), which is required to make a microemulsion. Possible causes include a mismatch between the surfactant ratios (Tween-80/Span-60), or the presence/absence of the co-surfactant agents (glycerol/ethanol, for these experiments glycerol), both of which can lead to fluid cloudiness, and consequently, decrease the ME stability. Some of the formulated MEs had transparent states (formulation numbers 17, 19, and 20).

**Figure 2:**
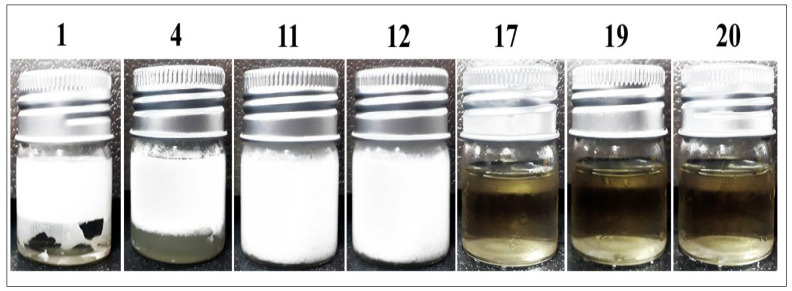
Photographed MEs, wherein formulation numbers 17, 19, and 20 are transparent.

According to the ongoing findings, the stability and physical appearances of the formulations were affected by the surfactant ratio, as well as also by the characteristics type and quantity of the co-surfactants used [25]. Since less surfactant was required for the preparations of transparent formulations, formulations 17–20 were chosen for further analysis. The fact that large surfactant concentrations are harmful, also played part in the selection of the surfactant. The handling, availability, and ease of practicality as well as cost-effectiveness of the MEs preparation was another crucial factor deciding the use of the combination to produce the required, optimal ME formulation (Figure 2).

### 2.2. Physico-Chemical Characterizations

**Figure 3:**
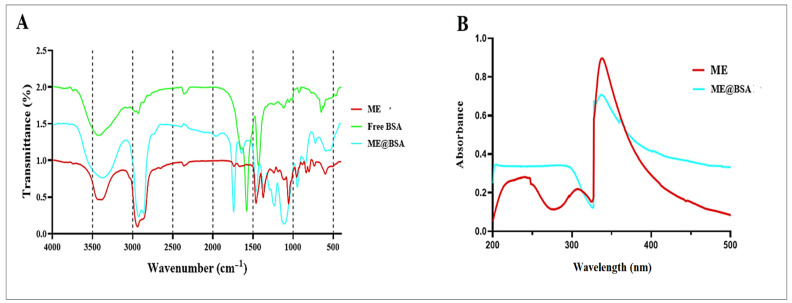
(**A**) FT-IR spectra of ME, free BSA, and ME@BSA, and (**B**) UV-Vis spectroscopy of the prepared microemulsion systems, ME and ME@BSA.

The chemical compositions and fabrication of the ME and ME@BSA were confirmed using FT-IR spectroscopy, as displayed in Figure 3A. The differences in the IR absorptions of the ME, BSA and ME@BSA were strikingly distinct (Figure 3A). The major differences in the absorptions were observed for the presence of strong CH stretchings, which were only present in the BSA and the ME@BSA at around 2900 cm^−1^. Other differences in absorption patterns were mostly confined to the absorption ranges between 1000 cm^−1^ and 1700 cm^−1^. The ME system showed peaks at 3400, 2900, 1400, 1300, and 1050 cm^−1^ for OH, CH stretchings, and ethereal bonds. The ME@BSA exhibited IR absorption peaks at 3400, 2900, 1750, 1400, 1250, and 1050 cm^−1^ for the presence of strong NH and CH stretchings, intense C=O and CH bendings, and ethereal C-O bonds, respectively, confirming the presence of the BSA in the ME@BSA formulation. The BSA exhibited absorption peaks at 3450, 2900, 1550, and 1400 cm^−1^ for the presence of strong NH, weak CH stretchings, amide bonds, NH bending peak, and CH bendings, respectively. The strong CH stretching and CH bendings, and C-O ethereal peaks, respectively, at 2900 cm^−1^ and between the 1050–1400 cm^−1^ absorption ranges indicated the presence of surfactants and the oil phases in the ME and ME@BSA formulations. These findings are consistent with the results obtained from several other reported studies. Swaidan, et al. [26] reported the preparation of a Cu@BSA nanocomposite, and the chemical composition of BSA was found to exhibit similar absorption peaks pattern. Moreover, the data from another study also indicated that the pattern of absorption peaks for a synthetic cholesterol-conjugated BSA system, matched the chemical composition of the BSA, as also confirmed in the current study. Finally, all characteristic absorption peaks regarding the ME system and BSA were observed in the ME@BSA’s FT-IR spectra, which indicated that the ME successfully ingrained the BSA, denoted as ME@BSA. The UV-Vis absorption spectra of the ME and developed ME@BSA systems were studied, and the absorption maximum is very slightly red-shifted with BSA presence. As shown in Figure 3B, adding BSA to the MEs enhanced the absorption intensity, and the maximum wavelength showed a slight red shift at around the ~340 nm wavelength for the product. 

The UV absorption intensity increased with the ME@BSA; perhaps the complex is formed by the amino acid residues of the BSA and the surfactant/co-surfactant in the ME system’s compositions, as reported in previous studies [27,28]. 

**Figure 4:**
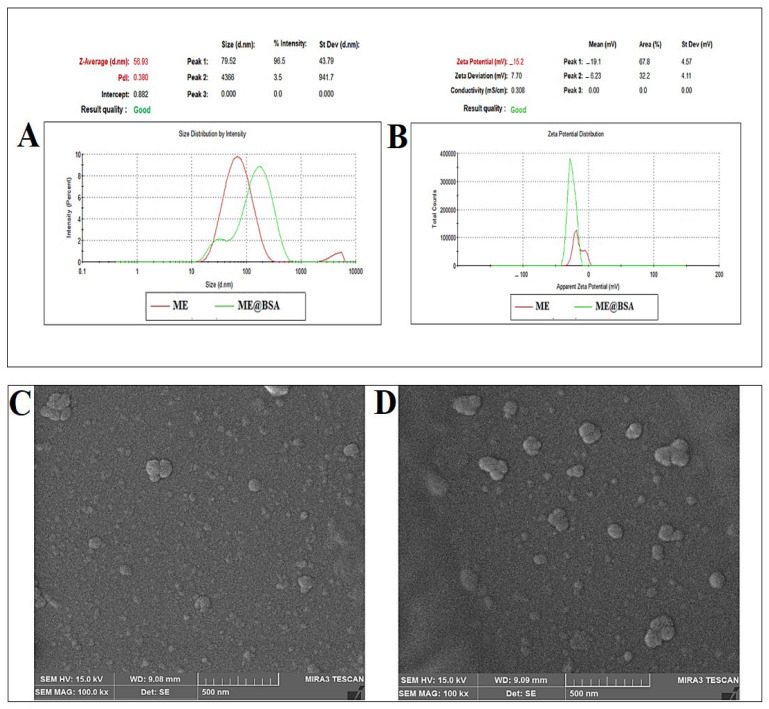
(**A**) DLS-based size distributions for the ME and ME@BSA; (**B**) ζ-potential of ME and ME@BSA; (**C**) electron micrograph (FE-SEM) showing the ME formulation, and (**D**) FE-SEM micrograph of the formulation ME@BSA.

A dynamic light scattering (DLS) method was also used to determine the hydrodynamic size of the ME@BSA. Figure 4A demonstrated that the ME and the ME@BSA had average sizes of 57.14 ± 0.25 nm, and 103.60 ± 3.36 nm, respectively. These results are due to the utilization of BSA, which caused an increase in the size of the ME@BSA, as compared to the ME formulation alone. Likewise, the charged surface of all the samples were predicted by zeta potential measurements. As obtained, the ME had a −14.73 ± 0.45 mV charge, while the ME@BSA had −24.23 ± 1.15 mV charge (Figure 4B). The electronegativity of the amine (NH) groups present in the surfactants, co-surfactants, and BSA, as well as the presence of hydroxyl, amide, and carboxyl functional groups, all contributed to the higher negative charge values of the zeta potential. Since the MEs were modified by BSA, the suggested system had a greater zeta potential, as also previously reported [29]. The high negative zeta potential values observed may have provided high physical stability, owing to the electrostatic forces dampened interactions in the water media for the formed formulations, the ME and ME@BSA. However, despite the changes in size, over time, upon storage, there was insignificant impact on the microemulsion properties, as evidenced by the good thermodynamic stability, and the system’s structural integrity (Figure 4).

**Figure 5:**
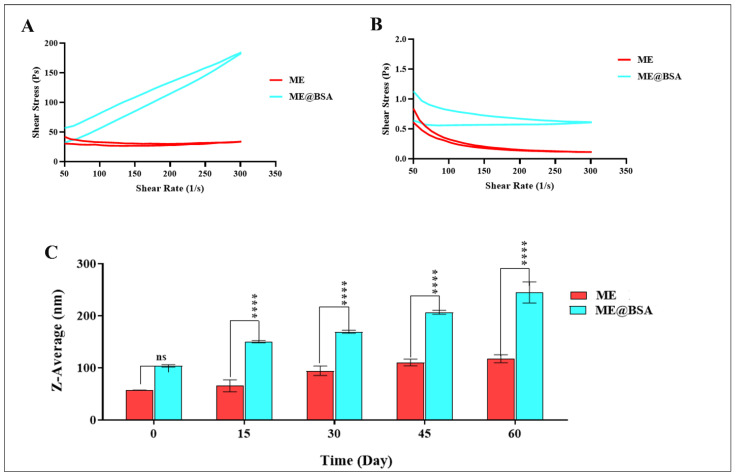
Rheological behaviors (**A**,**B**) of ME and ME@BSA; (**C**) Stability curves of ME and ME@BSA; **** meant significant effect at *p* < 0.0001, and, “ns” indicated no significant effect.

In addition, the FE-SEM electron micrographic analysis confirmed the formation of an ME, and ME@BSA (Figure 4C,D). These results are in consistent agreement with previously reported study [30]. The shear stress and viscosity results are shown in Figure 5. The shear rate of the ME under standard conditions (RT and normal atmospheric pressure) showed a sharp increase in the ME@BSA, nonetheless the ME did not show any noticeable change (Figure 5A). The viscous flow behavior of the ME and ME@BSA were demonstrated to be related to the apparent yield stress, caused by the interactions between the BSA and the continuous, or external aqueous phase. It can also be seen from Figure 5B that the ME is fluidic, perhaps Newtonian, while they were transferred to a pseudoplastic liquid state, when ingrained by the BSA and developed into ME@BSA formulation. Based upon these results, it appears that there are high levels of interaction between the surface of the ME system and the BSA [31,32]. Additionally, several researchers noted that the interactions between the BSA and the aqueous phase may also enhance the stiffness [33]. This means that the combination of BSA in the ME resulted in a more viscous end product. Additionally, when ME was ingrained by BSA in the aqueous medium (continuous phase) as a polar component, the aqueous medium became more viscous, and a new internal phase was formed. The incorporation of an internal phase increased the viscosity of the dispersion, especially at low shear rates, as illustrated in Figure 5B.

Furthermore, the hydrodynamic average size of the ME and ME@BSA, which were stored for 60 days, as a critical factor to predict the stability and potential aggregations, showed detectable increases in the size of ME and ME@BSA. However, no property changes were observed. Thus, it can be concluded that the suggested MEs were characteristically stable. These observations may have been due to the exceptionally low interfacial tensions between the oil and the water phases and the MEs, which exhibited excellent thermodynamic stability [34].

### 2.3. In Vitro and In Vivo Assays

**Figure 6:**
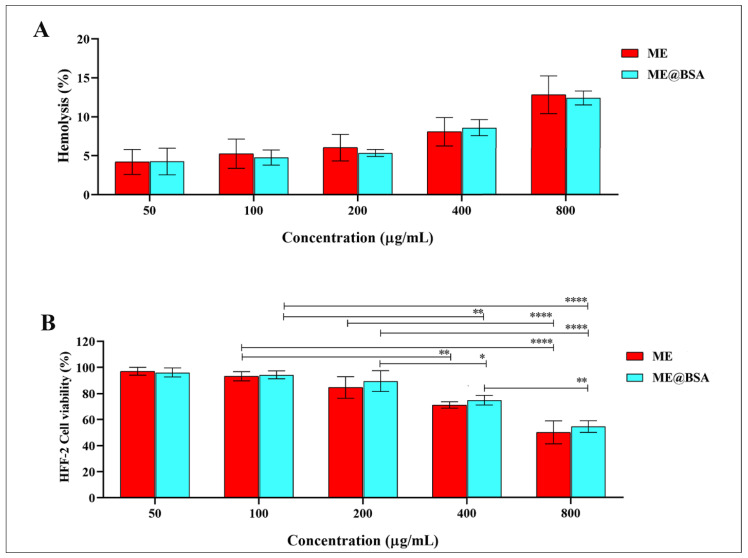
(**A**) Percentage of hemolysis induced by ME and ME@BSA at various concentrations and 37 °C temperature condition; (**B**) cell viabilities of the ME and ME@BSA on the HFF-2 cell lines. Data are represented as mean ± SD (n = 3) (* *p* < 0.05, ** *p* < 0.01, and **** *p* < 0.0001), and are considered as having significant, or no significant differences, respectively.

MEs have been implanted for their use in biomedicine, where they have direct contact with internal organs, tissues, and cells, and therefore their biological compatibility needs to be ascertained [35]. The proposed MEs are hemo-compatible, as they have negligible hemolytic effects on the human RBCs. The blood-based hemolytic activity was measured quantitatively at concentrations ranging from 50 to 800 µg/mL by evaluating the absorbance of the supernatant at 540 nm, with an Eppendorf Bio Photometer 17® (hemoglobin). As shown in Figure 6A, both the ME and ME@BSA, exhibited hemolytic activities between 4.21 ± 1.60% and 12.82 ± 2.40%, and 4.27 ± 1.70% and 12.42 ± 0.89%, respectively. Compared to the ME, the ME@BSA demonstrated a relatively lower hemolytic rate. A rising surfactant concentration, seems to have caused an increment in the hemolytic rate, as according to Feng, et. al. [36]. In addition to evaluating the biocompatibility of the system, the MTT assay was used to pretreat HFF-2 cells for 24 h with ME@BSA at 50, 100, 200, 400, and 800 µg/mL, which dramatically reduced the cell viability (Figure 6B). The MEs showed no significant toxicity to HFF-2 cells up to a concentration of 200 µg/mL as like normal cell lines, while having a statistically significant (*p* ˂ 0.001) decrease in the percentage cell viability at 800 µg/mL. As a result, this concentration was thought to cause cell death in HFF-2 cells, which can be explained by the HFF-2 cells’ oxidative stress-mediated cytotoxicity. Some researchers have also documented the cytotoxicity of ME@BSA on HFF-2 cells [21]. The ME@BSA is thought to induce intracellular ROS production in the HFF-2 cells, resulting in oxidative stress, and hepatotoxicity elicitations [37]. A more in-depth analysis of the importance of the current findings revealed transparent isotropic ME formation through the liquid combinations of oil, water, and surfactant, frequently in conjunction with a co-surfactant in the presence of BSA, was a more viable formulation. The dispersed phase droplet size in an ME system was under 100 nm. The MEs, which are proven to improve medication absorption, when applied topically, proved the point through the ROS controls. This could be attributed to the delivery carrier, which is typically constituted of the saturated, or unsaturated fatty acid components, and served as the oil phase. The in vitro cutaneous permeability of the diclofenac sodium, which crossed the skin surface, was examined using ternary combinations, and microemulsions preparation and characteristics studies [38]. Soybean lecithin ME gel was also explored as a suitable transdermal matrix for diclofenac and indomethacin deliveries [39]. 

**Figure 7:**
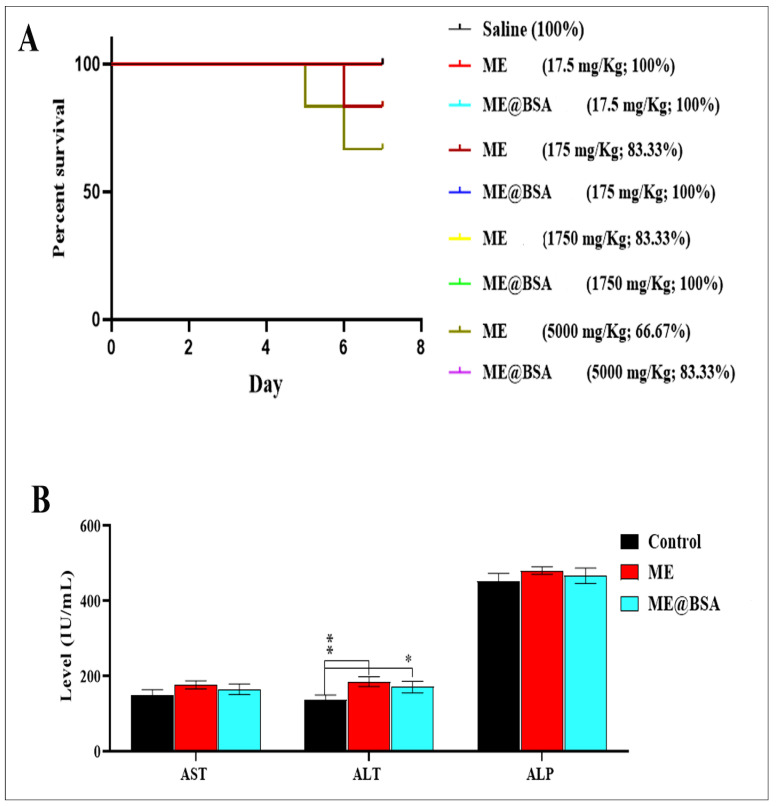
(**A**) Determination of LD 50 by administering increasing doses of ME@BSA, (**B**) Determination of the effect of ME and ME@BSA on the levels of AST, ALT, and ALP enzymes. * *p* < 0.05, and ** *p* < 0.01 were considered as significant.

In vivo acute oral toxicity assessments were also conducted to verify the effects of the selected ME’s intake in mice. The percentage of survival rate and physical behaviors were monitored after mice were orally fed the doses at 17.5–5000 mg/Kg range (Figure 7A). According to the data, 100–83.33% of mice fed under 5000 mg/kg ME, survived in all groups, while total 100% of mice fed with ME@BSA survived in all groups. Additionally, 66.67% and 83.33%, respectively, of the mice that were fed 5000 mg/kg ME, and ME@BSA, survived. It can be concluded that the suggested delivery carrier (especially ME@BSA) are grade 5 of the globally harmonized classification systems (GHS), and have LD_50_ values greater than 5000 mg/kg in mice.

Mice were used to evaluate the liver toxicity effects of the MEs and ME@BSA. The animals were sorted into three groups (five animals per group) denoted as ME, ME@BSA, and the control groups. As shown in Figure 7B, the mean ± SD values of the serum levels of ALT and AST of all the groups were <200 IU/L. Moreover, significant differences (*p* ˂ 0.005) were observed in the ALT levels, while the serum levels of the ALP were higher in all groups, but no significant differences (*p* ˂ 0.005) were observed. Cantarovich, et al. [40] found a statistically significant link between the dose of ME and its effectiveness. Moreover, Rahdar et al. [41] showed that a dose-dependent increase in serum liver enzymes is associated with microemulsion-mediated hepatotoxicity. It was also shown that taking microemulsion medications, reduced the liver ALT and AST enzymes level. The MEs have some limitations, including early drug leakage and release, as well as phase inversion, which suggested a need for the development of an efficient, perhaps more complex and differentiated MEs, which also provides longer holding time for payload, as well as take comparatively longer time to release the payload material at the site, which can be achieved with a quality-by-design approach in the MEs preparations for obtaining the late-stage leaks of payloads from the deigned MEs. The fact that many potent surfactants and/or co-surfactants possess toxicity, and are unsuitable for use as pharmaceutical materials [42], prompted the study to take the choice of BSA as a safe encapsulation material. Natural products, that have recently been demonstrated to be effective anti-oxidants and anti-inflammatory agents for medications, have been frequently administered due to their low toxicity in liver cells and other biological sites [43]. The MEs with an extraordinarily large specific interfacial area have provided increased adsorption at the micro-droplet surface of the ME. The low-toxicity BSA could be dissolved and adsorbed on the surface of the micro-droplet at the same time, with the solubilization quantities continuously increasing during the preparation of the delivery vehicle. Surfactants added to the emulsion systems during the preparation of the ME systems contain dispersions of oil and water, and that have demonstrated particular benefits for the administration of poorly soluble medications, as well as in terms of covering up the unpleasant taste of the medicinal substances [44]. 

## 3. Materials and Methods 

### 3.1. Reagents and Materials

Chemicals, including polyoxyethylene-sorbitan mono-oleate (Tween-80, CAS # 9005-65-6), glycerol (CAS # 56-81-5), sorbitan mono-oleate (Span-60; CAS # 1338-41-6), ethanol (CAS # 64-17-5), and MTT (3-(4,5-dimethylthiazol-2-yl)-2,5-diphenyltetrazolium bromide), were purchased from Sigma Aldrich, Schnelldorf, Germany, while the sesame oil, and solvents were purchased from Emertat Chime (Tehran, Iran), and HFF-2 cells were obtained from the American Type Culture Collection (ATCC, Manassas, VA, USA).

### 3.2. Preparation of O/W Microemulsion

The O/W microemulsions were prepared using a mixture of the surfactant (Tween-80, and Span-60) together with co-surfactant (glycerol) that were combined with the BSA. The optimal formulation included the dropwise addition of 7.60%, *w*/*w*, sesame oil (as the oil phase) to the aqueous phase at 50 °C for 30 min; while vigorously stirring the mixture that contained 70.04%, *w*/*w*, distilled water (with 2%, *w*/*v*, BSA), 18.60%, *w*/*w*, Tween-80, 2.21%, *w*/*w*, Span-60, and 4.10%, *w*/*w*, glycerol. The microemulsions formation were carried out according to the procedure of Gharbavi, et al. [21], with certain modifications. 

### 3.3. Physicochemical Characterization

The physical and chemical characteristics, and/or composition of BSA, ME, and ME@BSA were determined using FT-IR, UV-Vis, DLS, FE-SEM, and viscometer instruments based analyses.

### 3.4. FT-IR Analysis

Each sample was evaluated by FT-IR (Bruker, Tensor 27, Borken, Germany) spectroscopic analysis using potassium bromide (KBr) disks that had been mixed and mechanically grinded at a weight ratio of 1:10, and delivered in plate condition manufactured from pressing under the mechanical pressure device available at the laboratory. 

### 3.5. UV-VIS Spectroscopy

The production of MEs and ME@BSA (microemulsion with BSA constituent) were analyzed using a UV-SPECORD 210 PLUS Spectrophotometer (Analytik Jena, Jena, Germany). The spectral resolution of this device was 1 nm in the UV-Vis range of wavelength of 200–800 nm.

### 3.6. DLS and FE-SEM Analysis

DLS (Dynamic Light Scattering) analysis required diluting 200 μL of each sample with 2 mL of distilled water (DW) to an absorbance of 0.07 ± 0.02 units at 633 nm in a sterile Malvern sample vial. After dehydrating, the samples were coated in gold and examined using a FE-SEM (MIRA3, TESCAN, Bruno, Czech Republic) with a 15 kV acceleration voltage, and a magnification scale of 100,000×.

### 3.7. Rheological Behavior 

The rheological characteristics of the ME delivery carrier was investigated with the help of a viscometer (AMETEK GB LTD T/A Brookfield, Essex, UK). After removing any extra material, the upper surface of the reading plate was coated with three milliliters of ME. The remaining sample was discarded. The analysis of the data was performed with software that was provided by the manufacturer. The prepared ME’s physical stability was confirmed by checking the particle size distribution with DLS every 15th day for 60 days at room temperature (RT) storage condition.

### 3.8. Hemo-Compatibility Assay

A hemolysis assay was used to assess the hemocompatibility of the delivery carrier by inducing the rupture of the RBC membrane with the consequent release of intracellular hemoglobin into the plasma. For this experiment, a standard protocol described previously [45] was used. A precipitate of the RBCs was obtained by centrifuging a sample of healthy human blood at 4000 rpm for 5 min. The red blood cell (RBC) pellet was cleaned after removing the plasma, and then diluting it to a tenth of their original concentration using sterile, ice-cold PBS. Afterwards, the lyophilized powder of the ME was tested at concentrations ranging from 50 to 800 µg/mL for their hemocompatibility. Sodium dodecyl sulfate (SDS) 1%, and phosphate-buffered saline (PBS) served as the positive and negative controls, respectively. A shaking water bath was used to incubate all of the solutions, and after incubating the samples for 4 h at 37 °C, the non-lysed RBCs were separated by centrifuging the sample at 4000 rpm for 10 min at RT. A 96-wells plate was then used to hold 200 µL of the hemoglobin discharged into the incubation solution, and a microplate reader was used to measure their absorbances at 540 nm. The total weight of the ME determined the concentration tested in the hemolysis assay, which was performed three times, with the percentage of the hemolysis calculated as follows:

$$\mathrm{Hemolysis}\;\%=\frac{A_{\mathrm{treatment}\;\mathrm{sample}}-A_{\mathrm{negative}\;\mathrm{control}}}{A_{\mathrm{positive}\;\mathrm{control}}-A_{\mathrm{negative}\;\mathrm{control}}}\times 100$$

For the equation, A_treated sample_ represented the average absorbance of the treated sample, A_negative control_ represented the average absorbance of the negative control sample, and A_positive control_ represented the average absorbance of the positive control sample.

### 3.9. Cell Culture and Cell Viability Tests

An MTT assay was selected to assess the prepared ME system’s effects on cell viability. HFF-2 cells were obtained from the American Type Culture Collection (ATCC, Manassas, VA, USA), and seeded at a density of 6 × 10^3^ cells/well in 96-wells plates for 48 h (80% confluency) in DMEM supplemented with 10% Penicillin/Streptomycin, and 10% heat-inactivated fetal bovine serum (FBS). The HFF-2 cells were grown in the incubator at 37 °C, 5% carbon dioxide, and 80% relative humidity.

In parallel to the control wells (without treatment), the cells were grown for 24 h in 200 μL of fresh culture media with five different doses ranging from 50 to 800 g/mL for all formulations. After incubation, the culture medium was discarded and cleaned with PBS, and 20 μL of MTT (4 mg/mL) solution was added to each well. After 4 h, the medium was discarded, and 100 μL of dimethyl sulfoxide (DMSO) was added to each well to dissolve the violet formazan crystals. Following 15 min of shaking, cell viability was determined by measuring the absorbance at 570 nm using the microplate reader (Spectra Max 190). Cell viability was measured as a percentage, relative to untreated cells (100% survival rate). The data are presented as the mean ± SD of the three separate measurements.

### 3.10. In Vivo Experiments

These experiments were carried out on C57BL/6N male mice, where (n = 33) 18 mice chosen for the LD_50_ assay, and 15 mice were chosen for the liver function assay. The mice aged 6–8 weeks, and acclimatized for two weeks before the beginning of the studies. The mice were housed in open-top polypropylene cages with autoclaved wood shaving bedding, in groups of up to five per cage. The temperature in the room was set at 24 ± 2 °C, with air changes ranging from 16 to 20/h, humidity at 56 %, and an artificial light cycle of 12/12 h. The experiments were approved by the Animal Use and Care Administrative Committee of the College of Veterinary Medicine, University of Al-Qadisiyah (Approval ID-2022.UAQ4), and were in accordance with the Guidelines of the U.S. National Institutes of Health (NIH Publication No. 86–23, revised in 1996).

The animals were observed at least once a day by experienced staff to meet ASPA’s legal requirements. Building maintenance systems constantly monitored all facilities, notifying staff immediately if there was an emergency, or fault. Some of the areas, and animal shelters were monitored by the installed webcam.

### 3.11. LD_50_ Assay

Different components utilized in preparing the MEs formulations, such as, sesame oil, Tween-80, Span-60, BSA, and glycerol have known harmful effects. One way to assess the acute toxicity (short-term harmful potential) of the prepared, and selected ME is the LD_50_ analysis. Adult mice were chosen for acute oral toxicity trial to investigate the safety of the optimally produced and the selected ME. According to the OECD guidelines, all mice with an average weight of 30–35 g were housed in optimum laboratory conditions [46]. The ME and ME@BSA were administered orally to every animal for the LD_50_ determination at doses ranging from 175 to 5000 mg/kg. Before administration, as well as one day, and seven days afterwards, all animals were weighed. Animals’ physical activity and behavioral changes were also noted throughout the test. If the animals were alive after 24 h period, best available care was provided. If any of these animals were to live longer, the LD_50_ would be higher than the dose ceiling, and the test was planned to be terminated.

### 3.12. Liver Function Tests

The animals were split into three sets of five: a control group that received no treatment, an ME group, and an ME@BSA group, each receiving both formulations doses at 100 mg/kg intraperitoneally for 14 days. Data from prior research were used to determine the doses, administration schedule, and injection methods [47]. The animals were anesthetized by ether at the end of the treatment, and blood samples were taken from the heart’s left ventricle. The blood samples were kept in the laboratory for 10 min before being centrifuged for 15 min at 5000 rpm.

### 3.13. Statistical Analysis

Statistical analysis of the data was conducted with the help of GraphPad Prism 8® software. Each experiment was conducted thrice, and results are presented in terms of means and standard deviations. To test the statistical significance of the generated curve, ANOVA with repeated measures (one or two ways), as well as spline/LOWESS analysis, were applied. Differences were considered significant at * *p* < 0.05, ** *p* < 0.01, *** *p* < 0.001, and **** *p* < 0.0001.

## 4. Conclusions

An easy to prepare, feasible in handling, and non-toxic formulations of microemulsion (ME)-based carrier system, and its bovine serum albumin (BSA) counterpart, i.e., ME@BSA, were prepared, and characterized. The carriers encapsulated the designated model encapsulating payload, i.e., sesame oil. These preparations characteristics and behaviors were confirmed through FT-IR, UV-Vis, size, zeta potential, FE-SEM, and rheological analyses. The sesame oil, which is almost non-toxic, and may have prospective uses in co-delivery, with capability for multifunctional systems preparations, was envisioned in the design and preparation of the delivery platform. After undergoing in vitro and in vivo cytotoxicity testing, the developed microemulsion systems, i.e., ME and ME@BSA, were found to be non-toxic and safe, which proved the safety of the microemulsion and its different constituents, at least at the tested dose levels. Nonetheless, due to lack of the observable toxicity, the developed ME@BSA was seemed to have potential for newer applications in the fields of biomedicine, particularly for in vitro and in vivo delivery applications, involving the co-delivery to the difficult-to-deliver anatomical sites, e.g., the brain, placenta, ovaries, prostate, kidney, and pancreas. The work may also pave the way for the effective preparation of ME@BSA, which can be used in the in vitro and in vivo conditions for certain drugs candidates as payloads, and their probable development as molecular probes suitable for diagnostic purposes. The method also has the potential to be scaled-up for larger-scale preparation of these types of formulations.

## Figures and Tables

**Table 1:** Oil, water, surfactants, and co-surfactants constituents’ ratio, and phase states for the MEs.

Formulation Number	Sesame Oil	Tween-80 (%, *w*/*w*)	Span-60 (%, *w*/*w*)	Glycerol (%, *w*/*w*)	Ethanol (%, *w*/*w*)	Water (%, *w*/*w*)	State
1.	9.50	19.91	-	4.10	-	66.49	Separation phase
2.	9.50	19.91	-	-	4.10	66.49	Separation phase
3.	8.60	19.91	-	5.00	-	66.49	Separation phase
4.	8.60	19.91	-	-	5.00	66.49	Separation phase
5.	8.60	20.81	-	4.10	-	66.49	Separation phase
6.	8.60	20.81	-	-	4.10	66.49	Separation phase
7.	8.60	17.60	3.21	4.10	-	66.49	Slightly cloudy, phases separation after 1 week
8.	8.60	17.60	3.21	-	4.10	66.49	Slightly cloudy, phases separation after 10 days
9.	8.60	18.60	2.21	4.10	-	66.49	Slightly cloudy, phases separation after 2 weeks
10.	8.60	18.60	2.21	-	4.10	66.49	Slightly cloudy, phases separation after 2 weeks
11.	8.10	18.60	2.21	4.10	-	66.99	Slightly cloudy, and stable
12.	8.10	18.60	2.21	-	4.10	66.99	Slightly cloudy, and stable
13.	8.00	18.60	2.21	4.10	-	70.00	Very slightly cloudy, and stable
14.	8.00	18.60	2.21	-	4.10	70.00	Very slightly cloudy, and stable
15.	7.75	18.60	2.21	4.10	-	70.25	Very slightly cloudy, and stable
16.	7.75	18.60	2.21	-	4.10	70.25	Very slightly cloudy, and stable
17.	7.60	18.60	2.21	4.10	-	70.40	Transparent
18.	7.60	18.60	2.21	-	4.10	70.40	Very slightly cloudy, and stable
19.	7.40	18.80	2.21	4.10	-	70.40	Transparent
20.	7.40	18.80	2.21	-	4.10	70.40	Transparent

## Data Availability

All relevant data is contained in the manuscript.
